# Biased cognitions and social anxiety: building a global framework for integrating cognitive, behavioral, and neural processes

**DOI:** 10.3389/fnhum.2014.00538

**Published:** 2014-07-22

**Authors:** Alexandre Heeren, Wolf-Gero Lange, Pierre Philippot, Quincy J. J. Wong

**Affiliations:** ^1^Laboratory for Experimental Psychopathology, Psychological Science Research Institute, Université Catholique de LouvainLouvain-la-Neuve, Belgium; ^2^Behavioural Science Institute, Radboud University NijmegenNijmegen, Netherlands; ^3^Department of Clinical Psychology and Psychotherapy, University of CologneCologne, Germany; ^4^Department of Psychology, Centre for Emotional Health, Macquarie UniversitySydney, NSW, Australia

**Keywords:** social anxiety, cognitive biases, affective neuroscience, experimental cognitive psychopathology, clinical psychology, anxiety disorders and cognitive bias modification, cognitive therapy, behavior therapy

Social anxiety is a common emotional experience that occurs in response to the perceived threat of evaluation from others before, during, or after social situations. When social anxiety reaches a high level of severity such that functioning is impaired and associated with considerable distress, we refer to it as Social Anxiety Disorder (SAD). With a lifetime prevalence ranging from 7.3 to 12.1%, SAD is the fourth most common psychiatric disorder (Kessler et al., 2005). SAD has an early onset and tends to follow a chronic and debilitating course if untreated (e.g., Hayward et al., 2008). SAD usually precedes other anxiety, mood, and substance abuse disorders (e.g., Randall et al., 2001; Lampe et al., 2003).

Although the personal and economic costs of SAD as well as its comorbidity with other disorders have been very well-documented, uncertainty remains regarding the etiological and maintenance factors underlying this condition. As highlighted by Hirsch and Clark (2004), a curious feature of this condition is that it persists even if most individuals with SAD in their daily life perform naturalistic exposure to at least some feared social situations on a regular basis. At a fundamental level, one possible explanation for the enduring nature of SAD may be the way socially anxious individuals process social information. Accordingly, cognitive theorists have argued that negatively biased information-processing may contribute to the maintenance of SAD (e.g., Clark and Wells, 1995; Rapee and Heimberg, 1997; Hirsch and Clark, 2004; Morrison and Heimberg, 2013). An information-processing bias reflects a general processing advantage for disorder-relevant information in a given cognitive domain (e.g., attention, memory, interpretation, mental imagery). Such biases would lead individuals with SAD to evaluate social situations as more threatening than they actually are, and in turn, contribute to the maintenance of the disorder (e.g., Clark and Wells, 1995; Rapee and Heimberg, 1997).

Since the development of maintenance models for SAD (e.g., Clark and Wells, 1995), cumulative evidence indicates that SAD individuals do indeed exhibit such biased cognitions (for a review, see Hirsch and Clark, 2004), and further research has begun to uncover the behavioral, cognitive, and neural correlates of these biases (e.g., Rossignol et al., 2012; Hattingh et al., 2013). Given this progress, researchers have recently started to probe the causal nature of such biased information-processing by directly manipulating these biases in the context of etiological and maintenance frameworks for SAD. Recent findings suggest that these biases do indeed play a crucial role in the development (e.g., Hirsch et al., 2006; Heeren et al., 2012) and the maintenance (e.g., Amir et al., 2009; Clerkin and Teachman, 2010; Amir and Taylor, 2012) of SAD.

The research advances to date have generated interest in the biases of SAD within the scientific and practitioners community. However, integrative advances for channeling all information into a unified account of the different cognitive biases operating in SAD have not been offered thus far. For this purpose, the present Research Topic brings together a number of opinions, perspectives, reviews, and original research that provides state-of-the-art updates on this thriving relation between biased cognitions and SAD. These contributions provide a much needed advance in the current conceptualization of the mechanisms underlying biased cognitions in SAD and set the stage for future research avenues by clarifying and bridging conceptual gaps between different areas. The 11 papers of this Research Topic reveal that the diversity of the methods and approaches used can tell us more than the study of either topic in seclusion. Several key themes can be identified.

First, Haller et al. (2014) have provided a comprehensive neurodevelopmental framework to understand the brain and cognitive mechanisms that lead to biased cognitions in SAD.

Second, two research papers have focused on the implication of working memory capacity that may underlie biased cognition in SAD: Moriya and Sugiura (2013) investigated the role of working memory capacity in the inhibition of goal-irrelevant information and the direction of attention to distractors, while Salemink et al. (2013) explored the moderating nature of working memory capacity on threat-related interpretative bias.

Third, two papers have shed light on the need to move beyond face- and word-stimuli in the assessment and conceptualization of cognitive biases. Indeed, most of the available evidence comes from research paradigms using faces or words as materials. Social information, however, is also conveyed through other channels, such as vocal and postural cues. In their paper, Gilboa-Schechtman and Shachar-Lavie (2013) reviewed the fundamental and applied additive value of integrating nonverbal social cues in SAD research. Compatibly, Peschard et al. (2014) proposed a cross-modal perspective to advance the understanding of cognitive biases in SAD.

Fourth, three papers have focused on the development of new research approaches to gauge the key features of SAD. Van der Molen et al. (2014) explored the neural foundations of anticipating and processing social-evaluative feedback using event-related potentials. Gilboa-Schechtman et al. (2014) examined both self-reported and acoustic (i.e., vibrations of the vocal folds during phonation and speech) reactions to exclusion, acceptance, and popularity induced by a participation in an online ball-tossing game in SAD. In a critical review, Schulze et al. (2013) questioned whether gaze perception is a suitable way to assess attentional biases in SAD.

Finally, three papers have highlighted the need to translate basic advances in cognitive biases repeatedly observed among SAD patients into new innovative neurocognitive interventions directly targeting these biases. Maoz et al. (2013) reported an attempt to develop a subliminal computerized attention bias modification training program. Rinck et al. (2013) reported the benefits of a computerized training program that directly modifies the avoidance tendencies away from smiling faces exhibited by SAD individuals. Finally, Pictet (2014) commented on the acute gain of promoting positive mental imagery in cognitive bias modification when treating SAD individuals.

In sum, this Research Topic illustrates without question how different scientific approaches lead to an important road map for researchers and practitioners in the field of cognitive biases in SAD. We hope that this Topic moves the field closer toward a global framework for understanding the cognitive biases in SAD.

## Conflict of interest statement

The authors declare that the research was conducted in the absence of any commercial or financial relationships that could be construed as a potential conflict of interest.
